# Scratching the structure of moral agency: insights from philosophy applied to neuroscience

**DOI:** 10.3389/fnins.2023.1198001

**Published:** 2023-07-19

**Authors:** Francisco Javier Castro-Toledo, Pablo Cerezo, Ana Belén Gómez-Bellvís

**Affiliations:** ^1^Plus Ethics, Elche, Spain; ^2^Miguel Hernández University of Elche, Elche, Spain; ^3^The European University of Brain and Technology (NeurotechEU), Elche, Spain

**Keywords:** moral agency, neurophilosophy, free will, consciousness, responsibility

## Abstract

This paper explores the intersection between neuroscience and philosophy, particularly in the areas of moral philosophy and philosophy of mind. While traditional philosophical questions, such as those relating to free will and moral motivation, have been subject to much debate, the rise of neuroscience has led to a reinterpretation of these questions considering empirical evidence. This has led to tensions between those who believe neuroscience can provide definitive answers to very complex philosophical questions and those who are skeptical about the scope of these studies. However, the paper argues that neuroscientists and philosophers can work together to generate major scientific and social advances. To contribute to bridge the gap, in this paper we expose the complexity of moral experience from a philosophical point of view and point to two great challenges and gaps to cover from neurosciences.

## 1. Mind the gap between the train (neuroscience) and the platform (philosophy)

It has been a commonplace for philosophers, from the Greeks to the present day, to reflect on different aspects of the human phenomena such as the nature of knowledge, consciousness, free will, the self. Oceans of ink have been spilt on these issues and, nevertheless, many of these reflections would become obsolete (e.g., doctrines concerning the independence of cognition from the brain) in the face of the appearance and revolution of neurosciences (Churchland P. S., 1988). Indeed, inherent to the progress of experimental sciences (i.e., biology, psychology, neurosciences, etc.) is the reinterpretation of classical questions such as those mentioned above, which, moreover, intersect directly with the sphere of morality, in particular, moral judgments (Churchland, 2008; De Brigard and Sinnott-Armstrong, 2022). This reinterpretation in light of empirical evidence, specifically with respect to what neuroscience can tell us about how decisions are actually made, is interesting and necessary not only for a more realistic approach to the phenomenon by philosophy of mind and moral philosophy in particular (Bickle, 2009), but also because it can have an obvious impact on how we understand how we shape our own society and exist in it (Bechtel and Huang, 2022).

Consider, for instance, the practical application of questions of free will, agency, or moral motivation in the criminal justice system. This is a field where neuroscience is becoming increasingly relevant, both from an academic and a forensic practice point of view (Slobogin, 2017; Alimardani and Chin, 2019; Greely and Farahany, 2019; Noyon et al., 2019; Pernu and Elzein, 2020), precisely because the basic element of criminal responsibility is the free will, namely the freedom to decide to commit a crime, and one of the elements of the graduation of criminal responsibility rests on the moral motivation of the subject (Pernu and Elzein, 2020). To the extent that the criminal justice system is designed to hold accountable people who supposedly make decisions voluntarily and freely (which of course involves elements of conscience), it is logical that some of these assumptions are being affected by findings from neuroscience. Neuroscience studies could evolve concepts such as “voluntary action,” “intention,” “risk perception,” or “self-control” (Maoz and Yaffe, 2016), all of which are highly relevant for determining the existence of responsibility (Bunge, 2010). The question is not a minor one, because if empirical studies can show that, indeed, in a specific case the subject was not free or had no control over his actions as a consequence of the cognitive processes and abnormal functioning of his brain, the consequence is the impossibility of determining his guilt or, at least, of reducing it. This would also result in punishments that are more in line with the agent’s capacity to act at the time of committing the crime, thus not requiring him to act in a way that he cannot because of how his brain functions. In any case, it is also important to bear in mind two types of problems that neurosciences must face in order to be able to integrate more comfortably into questions related to law: on the one hand, the need to move from descriptive and correlational studies to studies of causality (Morse, 2022), as well as to design studies based on the key concepts in law to answer research questions of interest to this branch so that, in this way, they can have consequences in the field of regulation. On the other hand, and given the relevance and sensitivity of the field that the neuroscientific findings would affect, it is essential to take into account the limits and weaknesses of the approaches to address the elements that affect culpability from the neurosciences. Thus, for example, as Freedman and Zaami (2019) describe with respect to mental states, their determination relies solely on interviews with the accused, not on direct mental state or behavioral causation tests that are available.

It is also important to refer to an area of connection between philosophy and neurosciences that should be especially nurtured by philosophers, or rather neurophilosophers, such as neurolaw and neurorights. While the former refers to the “legal use and governance of neuroscientific tools, concepts, and data” (Shen, 2021, p. 175), or if one prefers “the interdisciplinary field which links the Brian to law, facilitates the pathway to better understanding of human behavior in order to regulate it accurately through incorporating neuroscience achievements in legal studies” (Petoft, 2015, p. 53); the latter refers to the development of research under strict ethical standards and the respect and guarantee of rights such as mental integrity (Ienca and Haselager, 2016); the right to psychological continuity (Decker and Fleischer, 2008); cognitive freedom (Bublitz, 2013); or the right to mental privacy.

In this same example, one can glimpse the tensions that neuroscience has generated for philosophical conceptions of free will or moral motivation and intuit the dichotomization of positions on the usefulness of neuroscientific studies. At the risk of caricaturing the complexity of the question, on the one hand, there would be those positions that are radically optimistic about the results of research on the brain and how it performs its functions, and it would seem that knowledge in this respect could provide answers and close debates on many questions of philosophy of mind and moral philosophy, the most controversial being that which would cast doubt on the existence of free will. On the other hand, there would be those who would be skeptical about the scope of these studies, claiming an impossibility of denying the freedom of the individual, and thus denying determinism in decision-making. As Smith (2011, p. 1) perfectly summarizes: “Scientists think they can prove that free will is an illusion. Philosophers are urging them to think again.” However, one could agree that it is neither reasonable that the concepts that are handled in moral philosophy and philosophy of mind remain impervious to the scientific studies that concern them and could inform them, nor is it reasonable to think that neuroscience can solve or provide definitive answers to the whole spectrum of questions that correspond to the normative field such as, for example, reflection on right and wrong, good and evil, justice and injustice, or even on philosophical concepts themselves, which neuroscientists try to reduce in their expression and operationalize in order to make them manageable in empirical studies.

Indeed, one should not overlook the fact that the very empirical nature of neuroscience constrains both the ontological and methodological commitments of neuroscientists, determining not only the different objects of interest, but also the way of approaching them. It is because of this that it is possible to claim, without it being considered a criticism that affects the possibilities of integrating empirical knowledge about the brain into conceptual discussions, that the neurosciences’ approach to moral experience is, at the very least, partial. This partiality is clear in light of the findings that have been made in moral philosophy for decades (or centuries) and which have emphasized that moral experience is structurally more complex than what is shown in empirical studies, either because of a limited operationalization of abstract concepts that are difficult to define (Smith, 2011); either because the findings in neuroscience are considered to be correlational rather than causal, and thus not sufficient to dislodge strong, foundational philosophical concepts of, for example, responsibility (Husak, 2022; Morse, 2022); or because they are questions that are beyond the description of reality and belong more to the realm of normative prescription (Patterson, 2022).

Despite the above, it can hardly be doubted that neuroscientists and philosophers are intended to work together, not only because they share many research questions, but also because their symbiotic relationship can generate major scientific and social advances (e.g., see the BRAIN Initiative, or the one in which this paper is framed, NEUROTECH EU project, among many others). That is, neuroscientists can benefit from the work of philosophers because the latter are trained to formulate relevant hypotheses from sound theoretical frameworks and conceptual developments (Blakemore, 2003), while philosophers can benefit from empirical knowledge about the factual premises of their conceptualizations.

However, for this relationship to develop, it is necessary to overcome a series of barriers that make the relationship between philosophy and neuroscience not as friendly as it should be, thus contributing to the dichotomization and polarization of positions mentioned above. According to De Brigard and Sinnott-Armstrong (2022), these barriers are basically the following: (a) language, insofar as it may be difficult for a neuroscientist to attend to the literature in philosophy and vice versa because both insist on not making an effort to translate for the other; (b) lack of mutual respect, as philosophers tend to underestimate empirical efforts as simplistic or reductionist of conceptually much more complex questions; and empiricists in general and neuroscientists in particular do not always value philosophical contributions in the field that concerns us here, perhaps because they consider them abstract and not very close to reality, disregarding their complexity.

The main aim of this paper is to contribute precisely to the second of these barriers to collaboration between the philosophy of mind and moral philosophy and the neurosciences, trying not to forget in the course of the work the one related to language. To this end, in the following section we will deal with two of the basic elements that must be addressed in order to be able to speak of moral experience or agency: moral knowledge and moral motivation. This will allow us, in the next section, to point out two major objects of research for neuroscience that are of interest to and directly connected with philosophy, emphasizing their problems and blind spots. This will allow us to conclude that, although the current understanding of (and from) neuroscience is incomplete, neuroscience can help inform future philosophical theorizing about the structure of moral agency.

## 2. Libet did not read enough moral philosophy: recalling two traditional elements of the structure of moral agency

If there is a starting point for the relationship between neuroscience and philosophy as it is currently understood, it is undoubtedly Libet’s (1985) experiment. In the mid-1980s, Benjamin Libet published his work on the subjective perception of freely making the decision to move one’s wrist in relation to other events, showing that what appear to be completely free and voluntary actions are taken automatically even before people consciously set out to perform them. This experiment did not *per se* debunk the concept of free will, but it was the empirical basis for questioning it (Libet, 1985), something that subsequent experiments that continue to work along the same research lines have continued to do (Soon et al., 2008; Fried et al., 2011; Schultze-Kraft et al., 2016). However, the results of this study were soon called into question. The criticisms are of various kinds (i.e., methodological in nature due to the type of tools used, imprecision in approaching the measurement of the time of occurrence, difficulties in establishing a causal relationship, among others [see Strzyżyński, 2013]). However, those that possibly weaken his conclusions from the point of view of philosophy and moral judgments are those that refer to the ambiguous use of words and concepts such as “decision,” “intention,” “desire,” etc., Mele (2006) as proxies for something that from philosophy is known as something much more complex such as free will: a concept indissolubly linked to that of moral experience. In this sense, and to avoid falling into simplicities that waste the potential of neuroscience evidence, it is necessary to highlight the complexity of the structure of moral experiences, and to report on the conceptual diversity and frameworks from which to frame the moral agency that allows decisions to be made on the basis of moral judgments (May et al., 2022). To this end, we will now explore two of the minimal units necessary to understand the structure of moral agency: the epistemological dimension and the dimension of moral motivation.

### 2.1. If moral agency exists, what kind of knowledge operates in it?

The first question of relevance in the understanding of the experiences of moral agency is related to its epistemological aspects or, in other words, to the conditions of possibility of moral knowledge of the different agents. From an ontological perspective, more neglected by neurosciences, but nuclear for philosophy, the question of whether there is something that can be the object of moral knowledge is key to understanding people’s moral behaviors. Discarding in this paper those theological theses that make moral reality (e.g., values) dependent on the existence of God (Baggett and Walls, 2011), some philosophers have suggested that moral agency is based on the perception of non-natural aspects of the world that can only be grasped through a special moral intuition or sense capable of discriminating between right and wrong, just and unjust (Sidgwick, 1907; Ross, 1930). On this there are, principally, two opposing extremes. On the one hand, ethical theories claim that objective and universal moral principles can be derived from self-evident *a priori* axioms (e.g., utilitarianism, deontologism, social contract or others). On the other hand, particularistic moral theories claim that the moral rightness or wrongness of an act is intuited or perceived directly from the characteristics of a specific situation (Gewirth, 1988; Little, 2000). In both cases, while intuition or moral sense seems essential to achieve some moral knowledge, it is also true that this faculty is, on the one hand, fallible due to the possibility of disagreement between people and, on the other hand, educable or influenceable (Miller, 2004; Caviola et al., 2014; Mogensen, 2017).

In contrast to the non-naturalistic approach above, moral naturalism holds that moral facts are part of the natural facts of the world and, therefore, approaching moral knowledge should be no more problematic than approaching other kinds of knowledge of the natural world (Copp, 2004; Lenman, 2006). In both its weak and strong versions, naturalism holds that moral facts are determined by non-moral facts either through an identity relation (e.g., pleasure = good; pain = bad) or through non-identity dependence. In this context, the naturalized epistemology introduced by Quine (1971) is especially informative. This paradigm suggests that any form of rigorous epistemology must be conducted within the parameters of science (a place where neuroscience would feel comfortable) and renouncing the traditional project of explaining moral agency from aprioristic elements independent of (or not dependent on) the findings of science. While this idea has gained wide acceptance, the relationship between naturalized epistemology and morality is more complex. Hence, in reaction to these more puritanical versions of naturalized epistemology, “pragmatic naturalism” advocates the combination of belief in natural reality with the importance of the practical utility of concrete human behaviors in establishing truth criteria and conditions of knowledge, which also affects the moral sphere (Campbell and Kumar, 2013).

Under these parameters of naturalized epistemology, it is possible to claim that the neuroscience approach to moral experiences has a strong evolutionary character (Pinker, 2017; Price and Sikkink, 2021; Vozzola and Senland, 2022). The evolutionary thesis is based on the premise that human morality, like any other natural characteristic, originated and persisted primarily as an adaptation shaped by natural selection (Allhoff, 2003; Denton and Krebs, 2017). Its success as an approach is due to the fact that the Darwinian explanation is clearer, more parsimonious, supported by evidence, and does not need to postulate the existence of absolute or aprioristic moral truths as has been done from other traditional ethical paradigms (i.e., dentologicism or utilitarianism). That is, moral experience would not be based on the perception of transcendental elements, but is contingent, and could have been different under other evolutionary parameters. Moreover, some experiments in neurobiology reveal evidence that evolutionarily humans have two main pathways of thought: a fast and intuitive one guided by emotions, and a slower and more deliberate one (Greene, 2008, 2014). And it has been explained that while deontological moral judgment would be guided mainly by the first system, consequentialist judgment involves the second (for a critical view, see Berker, 2009; May et al., 2022).

Alongside the evolutionary thesis of morality, cultural evolution has played an important role in leading us from certain proto morals to the astonishing variability of today (Copp, 2004). This social dimension of moral experience has also been developed several times by neuroscience (Ames and Fiske, 2010; Rule et al., 2013; Lizardo et al., 2020). In this context, the problem of moral disagreement becomes particularly relevant, as it seems to suggest that moral norms depend on the culture in which they develop and may vary between different societies (Plakias, 2019; Bambrough, 2020; Rowland, 2020). That is, when someone from one culture disagrees with the moral practices of another, they are simply expressing what is morally acceptable to their own culture. This has been explained by claiming that people show agreement or disagreement in their moral attitudes, but that there are no moral ground truths about which they can be wrong, so these disagreements are often resistant to resolution because moral knowledge is seemingly impossible. However, in the presence of these forms of skepticism and relativism, there may be alternative explanations that are compatible with the possibility of objective moral knowledge. More specifically, evidence from both moral psychology (Haidt, 2007; Robinson and Darley, 2007) and cultural anthropology (Kinnier et al., 2000; Curry et al., 2019) has been gathered that reinforces the idea of the existence of a limited number of basic or core values that are cross-culturally adopted by most people. Examples of these are benevolence, justice, loyalty, respect for authority, personal purity, or freedom, among others. Indeed, it has been stressed that these values are not stable: they are interpreted and applied differently not only across cultures but also over time within cultures (Trommsdorff, 2020).

In short, moral knowledge would develop through a cultural evolutionary process and arise from primitive tendencies shaped by natural selection and cooperative efforts to live peacefully while competing for resources. However, for traditional moral philosophy, these approaches are hugely problematic in their implications. Street (2006) raises the debate and argues that, ultimately, in epistemological matters we must choose between the evolutionary explanation of morality or the possibility of fully objective moral knowledge in the manner of traditional moral philosophy. That is, between believing that objectivity is a property of moral knowledge or paying the price, at all levels, of giving up thinking of moral truths as having objective status.

### 2.2. What role do judgments play in moral motivation?

The second core element of the structure of morality that we will discuss in this section is the role of motivation in moral agency. Philosophers have attempted to explain the relationship between moral motivation and moral judgments or beliefs through two main approaches: internalism and externalism. Broadly speaking, moral internalism holds that moral judgments are intrinsically linked to the motives and emotions of agents. According to this approach, moral judgments cannot be completely objective or independent of people’s subjective motivations (Bromwich, 2016). In other words, internalism argues that there is a necessary connection between moral beliefs and the motivation to act accordingly. As Rosati (2016) rightly captures, the basic phenomenon of moral motivation can be expressed in the following terms: when P judges that it would be morally right to φ, she is normally motivated to φ; if P later becomes convinced that it would be wrong to φ and right to ψ instead, she is normally no longer motivated to φ and becomes motivated to ψ. For example, according to internalism, if someone believes that it is morally wrong not to keep a promise, then that person must have an internal motivation to avoid not keeping it. On the other hand, moral externalism argues that moral judgments can be independent of people’s subjective motivations (Weatherson, 2019). Externalism claims that moral judgments are objective and do not depend on the emotions or motivations of individuals. According to this perspective, moral judgments can be true or false regardless of whether or not individuals are motivated to act accordingly (Martins, 2021). Following the example used above, according to externalism, even if someone has no internal motivation to avoid breaking a promise, if that person believes that it is morally wrong to break it, then his or her judgment is true. In short, while internalism claims that moral judgments are intrinsically linked to individuals’ motives and emotions, externalism holds that moral judgments can be true or false independently of individuals’ motivations.

Certainly, the debate on moral motivation has moved beyond genuine philosophical fora and has seen the benefits of advances in experimental psychology in understanding and responding to some of the key challenges to this element of moral agency. In this regard, several philosophers have recently addressed questions of metaethics and moral motivation from the perspective of psychology and have argued that this work has implications for the nature of motivation in general and for the debate between internalists and externalists of motivation (for a rigorous review, see Rosati, 2016). Among others, it is worth mentioning the important work of Schroeder et al. (2010), who identified four possible theories of moral motivation: namely, instrumentalist, cognitivist, sentimentalist, and personalist. Generally speaking, the instrumentalist holds, in Hume’s sense (Macnabb, 2019), that people are motivated by intrinsic desires that lead to the formation of non-intrinsic desires to satisfy the former. The cognitivist, on the other hand, holds that moral motivation begins with beliefs about what actions are right, independent of pre-existing intrinsic desires. The sentimentalist sees emotions as playing a central role in moral motivation, and the personalist sees the source of moral motivation, as posited by the Aristotelian thesis, in good moral character, especially in the virtues (Hursthouse, 1999; Van Zyl, 2018). From a neuroscientific perspective, while the instrumentalist and personalist viewpoints have been defended for their compatibility, something different happens with the cognitivist and sentimentalist proposals (Schroeder et al., 2010). Furthermore, although these approaches take up the neurophysiological basis of motivation and analyze the interplay between experimental psychology and philosophy, ontological and epistemological assumptions differ about the nature of psychological states and their functional and causal roles in moral agency.

Roskies (2003) argues for a more integrative approach to moral philosophy and neuroscience, recognizing that no single theory can fully explain moral motivation and that further research is needed to better understand the complex interactions between the mind and the brain. In this context, “motive internalism” has been of relevance. This approach holds, in general terms, that moral judgments are intrinsically linked to the motivation to act in a certain way, i.e., that when we make moral judgments, it is because we care about acting in a certain way and vice versa. According to the author, this approach faces the dilemma of being either too weak a proposition and lacking philosophical interest, or strong enough to be philosophically interesting but demonstrably false. If the intrinsic character of internalism implies that the connection between moral belief or judgment and motivation is maintained because of the content of the moral judgment, this poses serious difficulties for explaining moral motivation in cases where the person has no corresponding motivation (Roskies, 2006). Given the relevance that the proposal of motive internalism may have for neuroscience, we assess some arguments for and against it below.

Prinz (2015) suggests that there is empirical evidence to support internalism if it is understood as a psychological thesis. The first argument is based on sentimentalism, which says that emotions are components of moral judgments. Prinz (2015) claims that this thesis is supported by several neuroimaging studies showing that people enter emotional states when making moral judgments, and that the induced emotions have an impact on moral judgment (Rosati, 2016). Prinz (2015) also offers other arguments in support of internalism, such as experimental evidence that seems to show that people consider emotions to be necessary for moral attitudes. This second approach has received harsh methodological criticism, pointing out that the evidence provided does not necessarily adequately support sentimentalism as these arguments only show what people think.

In another now classic work, Sinnott-Armstrong (2006) proposes a critique of motivational internalism, indicating that some brain-damaged patients use moral terms and appear to make sincere moral judgments, but they lack the motivation to act in accordance with them. These are patients with lesions in the ventromedial cortex, who despite retaining the ability to judge social situations appropriately and make moral judgments, have difficulty acting in accordance with social mores and appear to lack appropriate motivational and emotional responses. However, despite the empirical evidence against motivational internalism, it is unclear whether the data are sufficient to undermine this approach, as some versions of internalism may be consistent with data on ventromedial frontal damage patients. In particular, some research suggests that psychopaths have an impaired ability to distinguish moral violations from conventional ones, which has led some to conclude that they have impaired moral concepts. Alternatively, in the case of patients with damage to the ventromedial frontal cortex, it has been argued that people who show acquired psychopathy do not show moral deficits, but that their deficits in non-moral aspects of life simply manifest themselves occasionally in moral situations (Aharoni et al., 2012; Borg and Sinnott-Armstrong, 2013).

## 3. Old challenges and new opportunities for neuroscience of moral agency

In the previous section, we have highlighted that moral experience, that which can be called moral agency, and which would determine decision-making on the basis of moral judgments, is something much more complex than the starting points of neuroscience studies have made clear. The variability of positions and approaches is an indication of the numerous attempts in philosophy to find an answer to the question of moral experience, which must necessarily start from two minimum units: epistemological possibilities and moral motivation. The aim of presenting a map of relevant aspects from philosophy is not to conclude or to convey the idea that the question is so complicated and abstract that neurosciences have nothing to do with it, but rather the opposite: the aim is to try to build bridges that will make the symbiotic relationship between philosophers and neuroscientists more productive. For this reason, and in line with all that has been said so far, in this section we will deal with the great challenges that, in our opinion, lie ahead for neuroscience. In this way, pointing out some of these areas facilitates the task of locating gaps and allows for ideas about how new studies in neuroscience might inform future philosophical theorizing. There are many issues that could be addressed here see De Brigard and Sinnott-Armstrong (2022), but we will focus on two major classical challenges such as free will and consciousness.

### 3.1. Challenge #1: the limits of free will

When it comes to the question of whether or not free will exists, it is challenging to find a historical period in which this question has not received attention in philosophy (Maoz and Sinnott-Amstrong, 2022). However, what sets apart the ancient debates on free will among theological determinists, fatalists, and scholastics (Redmond, 2007) from the current ones is the incorporation of scientific advances in disciplines such as physics (Penrose, 2015), chemistry, biology, and neuroscience (Koch, 2009). The experimental sciences have provided empirical evidence that can help position different perspectives in the debate on free will, including determinism (Spinoza, 2018), libertarianism (Swinburne, 2013), compatibilism (Fischer, 1971, 2012; Frankfurt, 1971), incompatibilism (Van Inwagen, 1975), and indeterminism (Mele, 2008). Specifically, a deterministic position posits that all human actions, whether conscious or unconscious, are determined by external and internal factors following causal logic. Our actions are determined by a series of physical, chemical, and biological factors that lead to a specific action. For example, if a particular physical–chemical reaction occurs in our brain, we can accurately predict a certain action. According to this position, free will either does not exist or is merely an illusion (Smilansky, 2000), as our actions are causally determined by prior material or immaterial phenomena that trigger them. In this strict sense, it also implies that if all relevant causal factors and natural laws were known, all our actions and decisions could be predicted with absolute certainty (Laplace, 2012). Libet’s experiments, as mentioned earlier, and their subsequent extension by Wegner (2004), highlight the illusory nature of free will and emphasize that what we perceive as free or conscious decisions are more closely linked to neurophysiological mechanisms than to true volition. When considering the context of neuroscience, the discussion revolves around how brain and mental processes determine our actions and decisions. Research in these disciplines has provided evidence linking neural activities and mental states to our decision-making, which raises profound questions about the existence of free will. Neurosciences have made various contributions to explaining conscious decision-making and how it correlates with variations in individuals’ neural activity. Moreover, neuroimaging techniques such as functional magnetic resonance imaging (fMRI) have led to arguments suggesting that certain neural processes occur before conscious decision-making, which could support the significance of unconscious processes and challenge the absolute role that conscious decisions play in shaping the existence of free will (Soon et al., 2008).

Additionally, research on brain plasticity suggests that our past experiences and the environment in which we find ourselves can gradually shape our brains, potentially influencing our actions and decisions. Brain plasticity refers to the brain’s ability to alter its structure and function in response to stimuli and experiences. This implies that our actions and decisions may be a product of brain processes molded by our interactions with the environment over time (Draganski and May, 2008). Likewise, studies such as the one conducted by Haynes (2006) have raised the possibility of predicting people’s intentions and choices by observing patterns of brain activity. Using machine learning algorithms, the researchers successfully decoded information from brain activity, accurately predicting whether a participant would make a left- or right-handed decision in a button-pressing task (Haynes, 2006). This, once again, raises questions regarding genuine autonomy and freedom of choice in our actions.

The counter-response to the deterministic stance comes from libertarianism. In brief, the libertarian position on the problem of free will posits the existence of a true capacity for free and indeterminate choice. According to this view, our actions and decisions are not entirely determined by causal factors of either a material or immaterial nature. Instead, we possess the ability to choose among different options without being fully conditioned by God, the universe, natural laws, the brain, or dynamics (Gabriel, 2019). This position implies that we are free agents capable of making autonomous and responsible decisions. On the other hand, proponents of free will from various fields argue that the current limitations of neuroscience and our understanding of brain processes are insufficient to dismiss the existence of genuine free will (Mele, 2006; Nahmias, 2011; Mele, 2014). They contend that even if correlations between brain activity and our decisions can be identified, it does not necessarily mean that our decisions are entirely determined by causal factors. Furthermore, some authors argue that this interpretation is based on a materialistic and reductionist conception of the brain (Gabriel, 2019) and relies on what is known as the mereological fallacy (Bennet and Hacker, 2003)–attributing to the brain, its parts, or components what corresponds to the subject as a whole.

On the other hand, a line of argument in favor of libertarianism that has gained interest in recent decades is based on the idea of quantum indeterminacy. According to this perspective, quantum processes occurring at the neural level can introduce randomness into the functioning of the brain and, therefore, into our actions and decisions. In this sense, quantum phenomena, such as the release of neurotransmitters or biochemical reactions, could have unpredictable effects that are not completely determined by known causal laws (Stapp, 2007). In addition, some studies in neuroscience, such as those carried out by Eccles (1986), have explored the possible relationship between brain processes and quantum indeterminacy, proposing that the release of neurotransmitters at the synapse could be influenced by quantum events, which would allow for an active intervention of the mind in the decision-making process. Thirdly, the incompatibilist position on the problem of free will maintains that genuine freedom is incompatible with determinism. This position can be divided between those who lean toward a deterministic stance and those who lean toward a libertarian stance. In the view of incompatibilists, if we inhabit a universe where all actions and events are determined by prior causes, true free will becomes impossible, necessitating a choice between one or the other. Within the incompatibilist position, authors such as Pereboom (2001) have proposed what is known as strong incompatibilism. Strong incompatibilism asserts the complete incompatibility between free will and determinism, contending that if our actions are determined by prior causes, we cannot be genuinely free in the traditional sense. According to this perspective, even if it is argued that we do not reside in a deterministic world, we would still need to consider factors such as genetic influences, environment, and upbringing that condition and restrict our ability to choose. Furthermore, even if determinism were false and some level of indeterminism exists in the world, the presence of indeterminism alone would not guarantee the existence of genuine free will.

In contrast to incompatibilism, the compatibilist position argues that freedom and determinism can coexist harmoniously. According to this viewpoint, free will does not necessitate complete indeterminacy or exemption from causal influences. Instead, it implies that we can act in accordance with our internal beliefs, desires, and motivations. Compatibilists contend that true autonomy and responsibility lie in our capacity to make rational decisions based on our own will, free from external coercion. They maintain that even if our choices are influenced by causal or deterministic factors, our ability to act in alignment with our values and desires reflects genuine freedom (Dennett, 1997, 2003, 2015; Hume, 2000; Gazzaniga, 2012). In the context of neuroscience, the discussion surrounding the compatibilist position revolves around how advancements in understanding the brain can potentially shape our conception of free will. Within the compatibilist framework, one line of argument suggests that neuroscience can elucidate the brain’s mechanisms and processes underlying our actions and decisions, without negating the possibility of compatible free will. Although correlations can be established between brain activity and our conscious and unconscious choices, this does not imply that our actions are entirely determined by causal processes or predetermined by external or internal factors. Furthermore, from a compatibilist standpoint, neuroscience may provide an empirical foundation for comprehending the development and shaping of our capacity for choice over time, such as through learning processes, brain plasticity, and environmental influences. However, this does not preclude the existence of compatible free will (Fischer, 1998; Vargas, 2013). In fact, these environmental and biological influences may constitute integral components of our ability to make autonomous and responsible decisions (Mele, 2009).

Lastly, in relation to indeterminism in the problem of free will, we would argue that our actions and decisions are not entirely determined by predictable or causal factors. This perspective acknowledges the existence of events or processes in the universe that are inherently random, opening up the possibility of free and indeterminate choices. These interpretations primarily stem from fields such as quantum physics (Penrose, 2015), probabilistic theories dealing with complex systems with numerous variables, and chaotic systems. Another line of argument for indeterminism is based on the notion that consciousness and subjectivity are fundamental aspects for the existence of free will. It posits that the subjective experience of consciousness and decision-making provides direct evidence that our actions are not entirely determined by external or internal forces (Chalmers, 1996). From this standpoint, free will cannot be solely explained by causal and deterministic processes but requires a deeper understanding of the nature of consciousness and subjectivity. Throughout this section, we have noted the distinctive characteristics, advantages, and disadvantages of each position. To carefully study and take a stance, it is crucial to consider the significance and impact of internal and external factors in decision-making, which may lead to the consideration that the existence of free will could be influenced by these factors in individuals’ normal course of action. However, it is important to recognize that despite advancements in these disciplines, especially in neuroscience, the question of whether free will exists and its implications for moral agency remains unresolved and still elicits uncertainty. Certain blind spots that significantly affect the results of empirical studies and their conclusions need to be addressed from the very design stage, particularly at the linguistic level concerning the diverse definitions of free will and moral agency contributed by philosophy (Mele, 2014; O'Connor and Franklin, 2022). Depending on the chosen frame of reference, the potential impact on free will vary. Additionally, the usage and interpretation of terms such as correlation, causation, and necessity must be carefully considered, as their application in different contexts may lead to different positions. Furthermore, methodological limitations pertaining to the contributions of various experiments and their relevance to free will should be highlighted. For instance, the ecological validity of experiments, which refers to the variation between the research context and the complexity of everyday life, plays a crucial role (Whittemore et al., 2001; Shadish et al., 2002). Moreover, questions have been raised regarding the capacity of neuroscience as a discipline to address the problem of free will and issues related to moral agency, such as the consequences of individuals’ actions from a legal, ethical, or moral standpoint. Finally, it is essential to acknowledge methodological limitations concerning our ability to attain a more accurate understanding of decision-making processes and their relationship to the brain. Given that the brain is a constant and evolving object of study, it appears premature and risky to propose a definitive neuroscientific answer to the problem of free will and, especially, the problem of moral agency.

### 3.2. Challenge #2: the nature of consciousness

Like the previous section, the questions surrounding consciousness, its components, its operation, its development, its relationship with our body, its existence, and its impact on moral agency have undoubtedly been among the most discussed problems throughout the history of philosophy. These questions have gained relevance in the field of philosophy of mind in recent years (Bickle, 2009). Furthermore, considering the potential impact of consciousness on moral agency, it is plausible to suggest that this is a multidisciplinary research area that significantly influences moral philosophy (De Brigard and Sinnott-Armstrong, 2022).

Although theories and developments on consciousness and its relationship with various fields of knowledge have existed since ancient times, albeit under different categories and names, it can be argued that the emergence of research in the field of neuroscience (such as neuroanatomy, neurobiology, cognitive neuroscience, neurophysiology, or psychiatry, among others) has provided knowledge that philosophical reflection alone could not encompass (Farah, 2010; Decety and Wheatly, 2015). While neuroscience cannot definitively answer the problem of consciousness and its relation to morality, it has offered a substantial empirical and theoretical framework to address old questions and, at both the theoretical and practical levels, raise new ones (Gazzaniga, 2005; Farah, 2010; Greene, 2014; Sahakian et al., 2015).

One of the empirical contributions of neuroscience to the discussions on consciousness pertains to research that explores the active brain regions during states of sleep or wakefulness (Duyn, 2012; Picchioni et al., 2013; Jorge et al., 2014; Mele et al., 2019). Some of these contributions stem from medical techniques such as non-invasive brain stimulation, including transcranial direct current stimulation (tDCS) and transcranial magnetic stimulation (TMS), which have provided insights into how the manipulation of neural activity can affect conscious experience. Modulating activity in specific regions has been observed to influence perception and conscious attention (Cohen Kadosh and Walsh, 2009). Similarly, neuroscience studies have addressed specific conscious processes like visual perception, decision-making, and self-awareness. These studies have identified distinct brain regions involved in each of these processes and have provided detailed information on how neural activity relates to conscious experience in these specific regions (Dehaene and Changeux, 2011; Seth, 2012). Additional advances in the field have come from research on REM or coma states following anesthesia, vegetative states, schizophrenia, or depression, which are states where consciousness may be altered (Alkire et al., 2008; Sanders and Maze, 2011; Mashour and Hudetz, 2018). In parallel, another extensively studied area in neuroscience, with potential implications for philosophical debate, is the study of brain lesions in different regions and their effects on consciousness.

For instance, lesions in the prefrontal cortex can affect attention (Churchland P., 1988), self-reflection, decision-making, and executive control. It has been observed that such lesions can result in alterations in self-awareness, emotional regulation, and action planning and execution (Stuss et al., 2001). These changes caused by brain-level lesions can manifest as difficulties in moral reasoning, impulse inhibition, goal setting, effective pursuit of goals, or language expression impairments like aphasia. Another critical brain region that has undergone extensive study is the thalamus, which serves as a gateway for sensory information to reach the cerebral cortex. Lesions in the thalamus can disrupt the transfer of crucial information and lead to disruptions in consciousness and vigilance states (Schiff, 2008). Patients with thalamic lesions may experience difficulties in maintaining wakefulness and alertness, resulting in drowsiness or even coma. Similarly, injuries to the brainstem can have a profound impact on consciousness, as the brainstem is responsible for vital functions such as sleep regulation, breathing, and environmental awareness. Brainstem injuries can lead to coma or a vegetative state, where the patient exhibits a lack of consciousness and basic motor responses (Parvizi and Damasio, 2003). In addition to these regions, lesions in other brain areas, such as the parietal lobe and temporal lobe, can also affect consciousness. The parietal lobe is involved in sensory integration and the perception of body space, while the temporal lobe plays a crucial role in memory and the recognition of objects and faces. Consequently, lesions in these specific brain areas can result in changes in the perception of one’s own body and autobiographical memory (Baars, 2003; Gainotti, 2013). It is this research, among others, that has prompted philosophers and neuroscientists to discuss the “neural correlates of consciousness” as the minimal neural mechanisms necessary for specific conscious perceptions (Metzinger, 2000; Tononi and Koch, 2008).

In this sense, research in this field aims to identify the neural correlates associated with consciousness production and uncover the principles by which biological phenomena give rise to the subjective states of sensation and alertness that characterize consciousness (Koch and Crick, 2001). It also seeks to determine whether consciousness is a global phenomenon of the entire brain or if it can be localized to specific regions, if at all. These scientific advancements, combined with the concepts explored in the philosophy of mind, lead us to consider various scenarios where moral agency could be compromised therefore. Particularly, this is relevant in the context of psychiatric conditions like schizophrenia (Northoff et al., 2011) or certain cases of autism spectrum disorder (Di Martino et al., 2014), individuals who have experienced brain injuries, or those affected by different chemical substances. These scenarios have significant implications, for example, in the field of criminal liability, where some individuals have exhibited criminal behaviors and attitudes due to brain tumors, which, upon removal, resulted in the disappearance of such behaviors (Maoz and Yaffe, 2016; Slobogin, 2017; Greely and Farahany, 2019). However, despite the valuable contributions of neuroscience, many questions remain unanswered, as this discipline alone cannot fully explain the complexities of consciousness. In this regard, we can highlight some of the challenges faced by neuroscience in this field. One central issue is the problem of subjective experience (Nagel, 1974), often referred to as the “explanatory gap” (Chalmers, 1995) or the problem of qualia (Armstrong, 1981; Chalmers, 2010). These concepts underscore the difficulty of establishing inferences between individuals because consciousness lacks an objective character in terms of experiences and their varying degrees. Similarly, there is the question of how subjective experiences, such as perceiving the color red, the taste of sweetness, or intense pain, arise from the brain’s physical activity. Although neuroimaging techniques and advancements in neurobiology have provided insights into the specific regions and areas of the brain that are activated and their action potentials, this does not constitute definitive evidence for the precise spatial localization of consciousness (Edelman and Tononi, 2000; Roskies, 2007; Klein, 2010) or an explanation of subjective consciousness. As Koch has stated, “Our understanding of the inner workings of the brain has not yet reached the level necessary to explain how consciousness emerges from the chemical and electrical activity of neurons” (Koch and Greenfield, 2007, p. 76). On another note, the quest to identify specific brain regions associated with the emergence of consciousness and/or related processes can be seen as a categorical error in Ryle’s sense. According to Ryle, mental concepts cannot be reduced or directly equated to physical concepts since they belong to different categories and play distinct roles in our understanding of the world. Thus, any attempt to reduce mental phenomena to purely physical explanations would be a categorical error (Ryle, 2000). Furthermore, from the field of philosophy of mind and philosophy of language, Kripke (1980) offers a valuable critique based on the concept of identity and necessity. Kripke (1980) argues that C-fiber stimulation is a physiological phenomenon responsible for transmitting pain signals in the nervous system. Traditionally, it has been assumed that there is a necessary and sufficient relationship between C-fiber stimulation and pain experiences, meaning that if there is C-fiber stimulation, there will necessarily be a corresponding pain experience, and vice versa. However, Kripke posits that hypothetical scenarios can be conceived where a person undergoes C-fiber stimulation but does not experience pain, such as in the case of specific surgical procedures. This challenges the necessary identity relationship between C-fiber stimulation and pain experiences, posing significant obstacles to a causal interpretation of necessity and sufficiency between a material substrate like C-fibers and a subjective experience like pain. These questions raise profound debates against an exclusively materialistic-reductionist interpretation of consciousness (Dennett, 1997; Edelman and Tononi, 2000). Similarly, as mentioned earlier, an adequate explanation is yet to be provided regarding how immaterial phenomena, such as consciousness, arise from a material substrate. An attempt to address these questions has emerged in recent decades from the philosophical position known as emergentism (Searle, 1992; Bunge, 2003). The emergentist position argues that the mind and consciousness are emergent features arising from the complexity and organization of the brain but cannot be solely explained in terms of physical properties and laws. It posits the existence of mental properties like subjective experience, intentionality, and thought that are distinct and cannot be reduced to the physical properties of neurons and synaptic connections occurring in our brains.

## 4. Final remarks

In this work, we have discussed the intricate nature of moral experience and its connection to moral decision-making. We propose that establishing stronger connections between philosophers and neuroscientists could enhance productivity across both fields. To contribute to this endeavor, we have focused on three significant challenges faced by neuroscience: the limits of free will, the nature of consciousness, and neurolaw. Regarding free will, we have analyzed the ongoing question of its existence and its impact on moral agency. It is crucial to consider the limitations in empirical studies, including linguistic variations in defining free will and moral agency, as well as methodological constraints regarding the ecological validity of experiments. The second challenge, the nature of consciousness, raises profound questions that have been debated throughout the history of philosophy. Within the study of consciousness in neuroscience, methodological limitations exist within the field itself and at the methodological level. These limitations should be considered by neuroscientists before embarking on new empirical studies. Notably, the lack of unity and consensus regarding findings and their interpretations impedes a definitive answer to the question of consciousness (Watt, 2004; Koch et al., 2016). Additionally, there is a prevailing inclination in neuroscience to align with specific theoretical approaches associated with materialism (Facco et al., 2017), which restricts exploration of alternative theoretical and methodological possibilities that could deepen our understanding of consciousness. Lastly, since consciousness encompasses diverse elements such as memory, language, thought, and perception, seeking to unify all these aspects under a single theory is overly simplistic. In conclusion, this paper emphasizes that recent advancements in neuroscience have enabled novel approaches to study these topics. We assert that the relationship between neuroscience and philosophy should be more collaborative, recognizing that there are still numerous unanswered questions to fully comprehend moral experience and decision-making. The integration of neurolaw into this discussion further raises ethical and moral concerns about the utilization of neuroscience within the legal system.

## Author contributions

All authors listed have made a substantial, direct, and intellectual contribution to the work and approved it for publication.

## Funding

This research has been developed in the framework of the project Neurotech^EU^ (https://theneurotech.eu/), Co-funded by the Erasmus+Programme of the European Union. Likewise, to the ‘Ayudas para la recualificación del profesorado universitario funcionario o contratado’ of the Ministry of Universities, the European Union and the Miguel Hernández University of Elche for 2021/2023.

In addition, this research is part from the project “Ius_Machina: On the normative basis and the real impact of the use of predictive algorithms in the judicial and penitentiary domains” (Ref: TED2021-129356B-I00), funded by MCIN/AEI/10.13039/501100011033 and by the European Union “NextGenerationEU”/PRTR.”

The authors declare that this study received funding from UNIVERSIDAD MIGUEL HERNÁNDEZ DE ELCHE. The funder was not involved in the study design, collection, analysis, interpretation of data, the writing of this article, or the decision to submit it for publication.

## Conflict of interest

The authors declare that the research was conducted in the absence of any commercial or financial relationships that could be construed as a potential conflict of interest.

## Publisher’s note

All claims expressed in this article are solely those of the authors and do not necessarily represent those of their affiliated organizations, or those of the publisher, the editors and the reviewers. Any product that may be evaluated in this article, or claim that may be made by its manufacturer, is not guaranteed or endorsed by the publisher.
